# Unexpectedly High Efficacy of SARS-CoV-2 BNT162b2 Vaccine in Liver versus Kidney Transplant Recipients—Is It Related to Immunosuppression Only?

**DOI:** 10.3390/vaccines9121454

**Published:** 2021-12-08

**Authors:** Paulina Nazaruk, Marta Monticolo, Anna Maria Jędrzejczak, Natalia Krata, Barbara Moszczuk, Joanna Sańko-Resmer, Tomasz Pilecki, Arkadiusz Urbanowicz, Michał Florczak, Leszek Pączek, Bartosz Foroncewicz, Krzysztof Mucha

**Affiliations:** 1 Department of Immunology, Transplantology and Internal Diseases, Medical University of Warsaw, 02-006 Warsaw, Poland; tapaulla@wp.pl (P.N.); marta.monticolo@gmail.com (M.M.); anamarie.jedrzejczak@gmail.com (A.M.J.); nkrata@wum.edu.pl (N.K.); tomaszpilecki@wp.pl (T.P.); arcurbi@gmail.com (A.U.); mflorek1983@gmail.com (M.F.); leszek.paczek@wum.edu.pl (L.P.); bartosz.foroncewicz@wum.edu.pl (B.F.); 2 Department of Clinical Immunology, Medical University of Warsaw, 02-006 Warsaw, Poland; bmoszczuk@gmail.com; 3 Department of Nephrology Nursing, Medical University of Warsaw, 02-007 Warsaw, Poland; joannnaresmer@poczta.onet.pl; 4 Institute of Biochemistry and Biophysics, Polish Academy of Sciences, 02-106 Warsaw, Poland

**Keywords:** BNT162b2 mRNA, COVID-19, kidney transplantation, liver transplantation, SARS-CoV-2, SOT, vaccination

## Abstract

The BNT162b2 vaccine is reportedly effective in preventing severe disease in more than 90% of the general population, but its efficacy in transplant recipients remains controversial. We aimed to determine the immune response to the BNT162b2 vaccine in kidney (KTRs) and liver transplant recipients (LTRs). In this retrospective cohort study, we included randomly 65 KTRs and 65 LTRs, who received two 30 μg doses of BNT162b2 vaccine in 3-to6-week intervals. We analyzed the anti-SARS-CoV-2 spike protein IgG antibody (anti-S1 Ab) titer, biochemical liver and renal tests, immunosuppressive drug trough level, and clinical follow up 4–6 weeks after the first dose and 4–8 weeks after the second dose. The level of protective antibodies was 57.1% in KTRs and 88.9% in LTRs after the second dose. The anti-S1 Ab response was significantly associated with sex, age, and history of COVID-19. A tacrolimus dose at vaccination but not its trough level was significantly correlated with the increase in anti-S1 Ab titer after the second vaccine dose in LTRs. Rejection episodes did not occur after vaccination. Our results showed a higher than previously reported humoral response to the BNT162b2 vaccine in KTRs and LTRs, which was dependent upon age, type of transplanted organ, and immunosuppression.

## 1. Introduction

Coronavirus disease 2019 (COVID-19) is caused by severe acute respiratory syndrome coronavirus 2 (SARS-CoV-2) and is the result of the complex interplay between viral dynamics and host immune responses [1]. Most end-organ complications that characterize severe COVID-19 are attributable to a dysregulated immune response that follows SARS-CoV-2 infection. In January 2021, two mRNA vaccines: mRNA-1273 and tozinameran (BNT162b2) were introduced to prevent COVID-19. Both vaccines are reportedly effective in preventing severe disease in more than 90% of the general population, across all age groups and in pre-specified subgroups at high risk of severe disease [2,3]. However, these trials excluded patients receiving chronic immunosuppressive therapy such as solid organ transplant (SOT) recipients.

There are several concerns about the use of SARS-CoV-2 vaccines in transplant recipients. First, theoretically, inducing alloimmunity might result in graft rejection. Vaccines could induce alloimmunity by stimulating previously alloreactive immune cells or through the non-specific stimulatory effects of adjuvants, which could lead to de novo alloimmunity. Indeed, such donor-specific antibodies (DSA) were previously detected in kidney transplant recipients (KTRs) and heart transplant recipients who received AS03-adjuvanted 2009 influenza A virus subtype H1N1 pandemic influenza vaccines [4,5,6]. Despite the potential reactogenicity risk, in clinical trials of both mRNA-1273 and BNT162b2 vaccines, there was no evidence of increased risk of rejection or autoimmune phenomena after the first dose [7,8]. Another concern related to mRNA vaccination is the virus-specific immune response and its duration. Pre-pandemic experience with licensed vaccines has shown that SOT recipients mount less robust immune responses to vaccines compared with non-transplant patients, regardless of the vaccine type [9]. The specific impact of each component of immunosuppression (IS) on vaccine immunogenicity is poorly understood. 

The first study evaluating the immune response of 23 KTRs to the standard two-dose regimen of BNT162b2, revealed that only 22% were positive for anti-SARS-CoV-2 spike protein IgG antibody (anti-S1 Ab) after the second dose [10], whereas vaccination was effective in almost 100% of healthy controls. Additionally, the mean anti-S1 Ab titer of KTRs was significantly lower. In another study, 80 LTRs developed a substantially lower humoral response to the BNT162b2 vaccine than controls [11]. However, LTRs show superior results compared to other SOT recipients reported so far [12]. Factors related to serological Ab responses include age, renal function, and type of IS used [11]. Lastly, a case was reported of a lung transplant patient who failed to develop neutralizing Ab to SARS-CoV-2 after two doses of the BNT162b2 mRNA vaccine [13]. These data confirmed the theory/observation that SOT patients may have an impaired immune response following mRNA-based SARS-CoV-2 vaccination. Patients with a history of COVID-19 might benefit from a different approach. The rapid increase in SARS-CoV-2 neutralizing Ab after the first dose of BNT162b2 vaccine in a group of previously infected individuals was reported. They reached titers similar to those observed in naïve subjects only after the second dose [14]. These findings raise the question of whether a single vaccine injection might be sufficient for patients with COVID-19 history. Indications to booster with the third dose of the SARS-CoV-2 vaccine are currently under discussion [15,16,17]. Moreover, the coronavirus cross reactivity in the context of response to potential viral mutations was recently addressed [15]. These issues seem to be particularly important for all immunocompromised and chronic disease patients such as SOT recipients.

Considering the aforementioned concerns, we performed this study to assess the serum anti-S1 Ab levels as well as graft function in KTRs and LTRs after BNT162b2 vaccination. 

## 2. Materials and Methods

### 2.1. Study Design

This was a retrospective cohort study of randomly chosen 65 KTRs and 65 LTRs, who -received two 30 µg doses of BNT162b2 vaccine at a dosing interval of 3-to-6 weeks. Patients were qualified by the designated staff at the Medical University of Warsaw—Infant Jesus Hospital vaccination center. The inclusion criteria were age over 18 years and stable graft function. Patients with a history of allergy to any vaccine, acute illness, or fever within 72 hours prior to vaccination, or any chronic medical condition considered progressive or uncontrolled and required hospitalization within the previous 3 months were disqualified from vaccination. A history of COVID-19 disease more than 2 months prior did not disqualify patients from vaccination.

As part of the standard follow up of patients after vaccination, anti-S1 Ab levels were measured during routine visits to the Transplant Outpatient Clinic: 4–6 weeks after the first dose and 4–8 weeks after the second dose. The clinical status of the patients and the biochemical parameters of the graft function were also assessed during these follow-up visits. 

We did not include healthy controls as the response rate to vaccination in the general population was previously reported. Common patterns of Ab response to four different SARS-CoV-2 antigens after vaccination of healthy individuals is known [18]. Additionally, measuring the post-vaccination titers of COVID-19, is not recommended [19]. Therefore, taking into account the existing data from healthy subjects, the lack of recommendations to control their antibody titer, and retrospective study design, we did not include the control group. Medical records were reviewed, retrospectively, for: (1) the immune response to the vaccination as measured by anti-S1 Ab titers at two time points: 4–6 weeks after the first dose and 4–8 weeks after the second dose; and (2) the function of the transplanted kidney or liver following both BNT162b2 doses, defined by kidney and liver blood biochemical tests, immunosuppressive drug trough concentration, blood cell count, and clinical follow up. The difference between anti-S1 Ab titer after the first and second dose of vaccination was presented as delta (Δ) anti-S1 Ab.

### 2.2. Biochemical and Clinical Tests

Routine biochemical tests including blood morphology, serum creatinine, alanine aminotransferase (ALT); aspartate aminotransferase (AST); gamma-glutamyltranspeptidase (GGTP), alkaline phosphatase (ALP), bilirubin, complement proteins, urine analysis and urinary protein excretion were carried out using automatic biochemical analyzers: Cobas Integra 400 plus (Roche Diagnostics, Mannheim, Germany) and Elecsys 2010 Roche. Tacrolimus and cyclosporine trough concentrations were assessed with use of Architect Analyzer i2000SR (Abbott, Chicago, IL, USA).

The glomerular filtration rate (eGFR) was estimated according to the chronic kidney disease-epidemiology collaboration (CKD-EPI) equation. Body weight in kilograms was divided by the square of height in meters (kg/m^2^) to evaluate body mass index (BMI).

### 2.3. Anti-S1 Ab Testing

The SARS-CoV-2 IgG II Quant test (Abbott Laboratories, Chicago, IL, USA) was used to determine anti-S1 Ab level by designated personnel in the hospital laboratory. This is an immunochemical test using a microparticle and chemiluminescent marker (Chemiluminescent Microparticle Immunoassay, CMIA). It is used for both qualitative and quantitative IgG Ab determination, including neutralizing antibodies against the receptor-binding domain of the S1 subunit of the SARS-CoV-2 virus spike protein in human serum and plasma on the ARCHITECT analyzer. The units used in the test are arbitrary units per mL (AU/mL). The analytical measuring interval ranged from 21.0 AU/mL to 40.000 AU/mL, the cut-off was 50.0 AU/mL, and results <50.0 AU/mL were considered negative and >50 AU/mL were positive. The relationship between Abbott AU/mL and binding Ab units per mL (BAU/mL), which is a standardized unit according to the World Health Organization [20,21], is presented by the following formula [22]:

BAU/mL = 0.142 × AU/mL

However, 14 of 130 patients were tested for anti-S1 Ab in the laboratories of their place of residence. Their results were obtained using other types of tests and presented in different units (AU/mL, BAU/mL, international units per mL (IU/mL), relative units per mL (RU/mL)). It was not possible to establish a reliable mathematical correlation between the measurements of anti-S1 Ab concentrations obtained in different units; thus, they were excluded from the statistical analyses. However, they were included in the summary of the vaccine anti-S1 Ab protective level, according to each testing method cut-off. Therefore, 116 SOT recipients were selected for the final analyses.

### 2.4. Anti-Nucleocapsid Protein Ab Testing

To assess the potential effect of COVID-19 history on anti-S1 Ab level, anti-SARS-CoV-2 nucleocapsid protein IgG antibodies (anti-N Ab) were measured using CMIA technology and estimated as a relative light unit (RLU) by the Abbott test on the Alinity i analyzer (Abbot Laboratories). Results are expressed as an index (signal/cut-off [S/CO]). The index cut-off point was 1.40 (S/CO), therefore the results <1.40 (S/CO) were considered negative and >1.40 (S/CO) were positive. This measurement was performed twice: 4–6 weeks after the first vaccine dose, and 4–8 weeks after the second dose along with the anti-S1 Ab (Appendix A, Table A4).

Statistical analyses were performed in R version 3.6.1. and Statistica 13.1 (StatSoft). Results are expressed as the mean ± standard deviation (SD), median (MD) ± interquartile range (IQR), or a percentage value. All variables were tested for normal distribution by the Shapiro–Wilk test. Non-normally distributed variables were analyzed by non-parametric tests (Mann–Whitney U test and Kruskal–Wallis test). The association between parameters was analyzed using Spearman’s correlation. *p* < 0.05 was considered significant. 

This study was approved by the Medical University of Warsaw Institutional Review Board (AKBE/182/2021).

## 3. Results

Between January and June 2021, 130 SOT recipients (65 KTRs and 65 LTRs) received 2 doses of BNT162b2 vaccine and underwent subsequent serologic testing for anti-S1 Ab. Positive response to vaccination was found in 58.5% of KTRs and 83.6% of LTRs. After exclusion of 14 individuals, who were tested in external laboratories with the use of incomparable methods, 116 patients (61 KTRs and 55 LTRs) were subject to further analyses. The mean age of KTRs and LTRs at vaccination was 54.4 years and 58.4 years, respectively; 54.1% of KTRs and 32.3% of LTRs were females. Depending on the transplanted organ type as well as the primary liver disease of the LTRs, they received mono-, double- or triple-drug maintenance IS consisting of: glicocorticosteroids (GS), azathioprine (AZA), mycophenolate mofetil (MMF), cyclosporine (CsA), tacrolimus (TAC), everolimus (EVR), or sirolimus (SIR). 

A history of COVID-19 confirmed by a positive SARS-CoV-2 PCR test was documented in 8.2% of KTRs and 9.1% of LTRs. Interestingly, none of the KTRs in contrast to 60% of LTRs developed loss of smell or taste as a symptom of COVID-19. Detailed patients’ characteristics are summarized in Table 1. 

### Immune Response in SOT Patients

**Figure 1:**
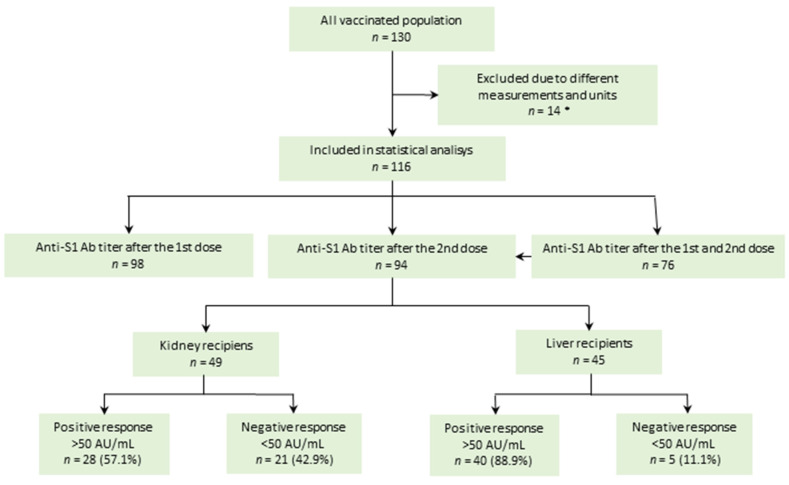
Enrollment flow chart and response to vaccination after the second dose of BNT162b2 vaccine. * Other measurement units of anti-SARS-CoV-2 spike protein antibody titer than AU/mL. Anti-S1 Ab = anti-SARS-CoV-2 spike protein antibody; 1st dose = 4–6 weeks after the first dose of vaccination; 2nd dose = 4–8 weeks after the second dose of vaccination.

The anti-S1 Ab titer results of 116 patients were obtained after the first and/or the second dose of vaccine (Figure 1). Early response to BNT162b2 (after the first dose) was observed in 44.2% of KTRs and 63% of LTRs. The response rate evaluated after the second dose was 57.1% in 49 KTRs and 88.9% in 45 LTRs. 

**Figure 2:**
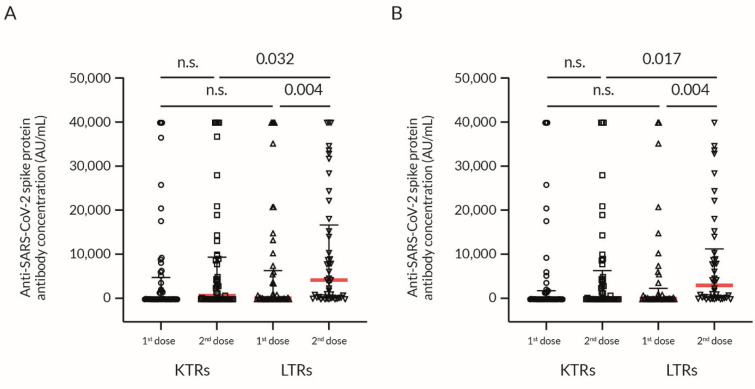
Anti-SARS-CoV-2 spike protein Ab production in response to BNT162b2 vaccination in KTRs and LTRs. Immune response to vaccination depending on the type of transplanted organ. Values are presented as the scatter-dot plot with median (middle line), lower (25%), and upper (75%) quartile (as whiskers); the *p*-value was calculated with the non-parametric Mann–Whitney U Test. (**A**) All patients including positive history of COVID-19 infection (confirmed by PCR test), (**B**) patients without confirmed COVID-19 infection. 1st dose = 4–6 weeks after the first dose of vaccination; 2nd dose = 4–8 weeks after the second dose of vaccination; BNT162b2 = BioNTech/Pfizer COVID-19 mRNA vaccine; KTRs = kidney transplant recipients; LTRs = liver transplant recipients; n.s. = not significant.

**Figure 3:**
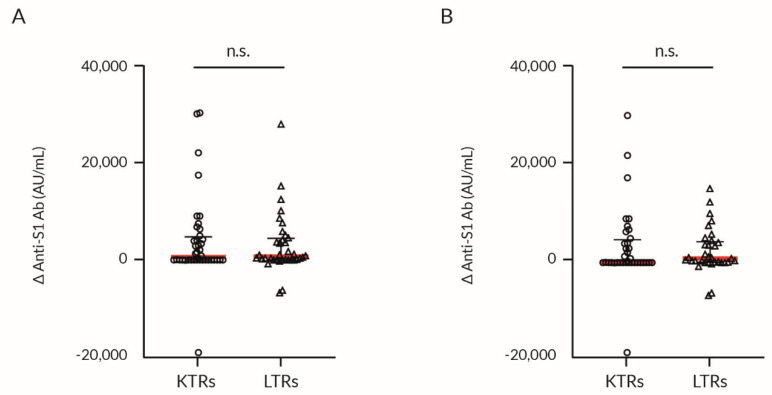
Anti-SARS-CoV-2 spike protein Ab production in response to BNT162b2 vaccination in KTRs and LTRs. Immune response to vaccination depending on the type of transplanted organ. Values are presented as the scatter-dot plot with median (middle line), lower (25%), and upper (75%) quartile (as whiskers); the *p*-value was calculated with the non-parametric Mann–Whitney U Test. (**A**) All patients including positive history of COVID-19 (confirmed by PCR test), (**B**) Patients without confirmed COVID-19. Δ anti-S1 Ab—difference in anti-SARS-CoV-2 spike protein antibody concentration between the first and the second dose of the vaccination; BNT162b2 = BioNTech/Pfizer COVID-19 mRNA vaccine; KTRs = kidney transplant recipients; LTRs = liver transplant recipients; n.s. = not significant.

LTRs produced a significantly higher anti-S1 Ab titer after the second dose of vaccine than KTRs (Figure 2A,B; Table 2); however, the difference between the Δ anti-S1 Ab in LTRs and KTRs was non-significant (Figure 3A,B). 

**Figure 4:**
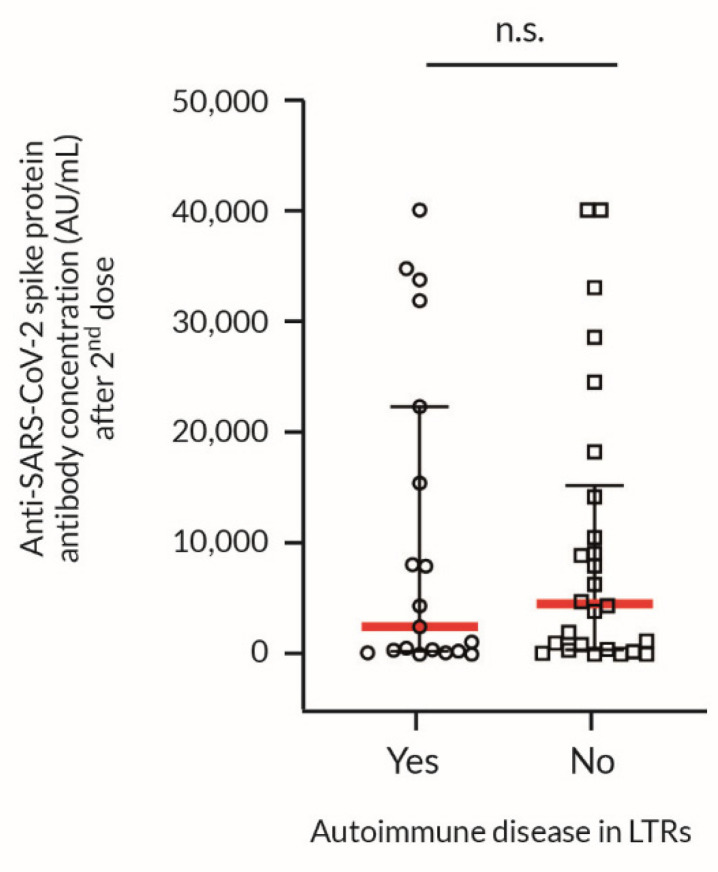
Anti-SARS-CoV-2 spike protein Ab concentration after the second dose in response to BNT162b2 vaccination in LTRs with autoimmune or non-autoimmune etiology of liver transplantation. Post-vaccination antibodies production in LTRs with autoimmune (autoimmune hepatitis, primary biliary cirrhosis or primary sclerosing cholangitis) and non-autoimmune causes of liver transplantation. Values are presented as the scatter-dot plot with median (middle line), lower (25%), and upper (75%) quartile (as whiskers); the *p*-value was calculated with the non-parametric Mann–Whitney U Test. 2nd dose = 4–8 weeks after the second dose of vaccination; BNT162b2 = BioNTech/Pfizer COVID-19 mRNA vaccine; LTRs = liver transplant recipients; n.s. = not significant.

The influence of the autoimmune comorbidities could be evaluated only in LTRs, since the data on primary kidney diseases in the majority of KTRs is unknown. Interestingly, we found that the coexisting autoimmune diseases in LTRs had no influence on the response to vaccination compared to those LTRs with non-autoimmune etiology (Figure 4). We did not find such observations in the literature.

A significant association was observed between the Δ anti-S1 Ab and patient age and estimated glomerular filtration rate (eGFR). This relationship with age was significant in KTRs only. The anti-S1 Ab titer after the first and second vaccine doses was also correlated with age, particularly in KTRs. A positive anti-N Ab test was significantly correlated with anti-S1 Ab titers after the first and second doses of vaccine. Interestingly, such correlation after the 1st dose was significant in KTRs and LTRs, whereas after the 2nd dose was significant in KTRs only. In addition, the TAC dose at vaccination was significantly correlated with anti-S1 Ab titer after the second vaccine dose in LTRs. Such a correlation was not observed in patients taking CsA. There were no associations between serum trough concentrations of TAC or CsA and Δ anti-S1 Ab titer (Table 3). We found that female LTRs had a significantly higher response to vaccination than female KTRs, both after the first and the second dose (Table 4). The quantity of anti-S1 Abs is summarized in Table 2.

**Figure 5:**
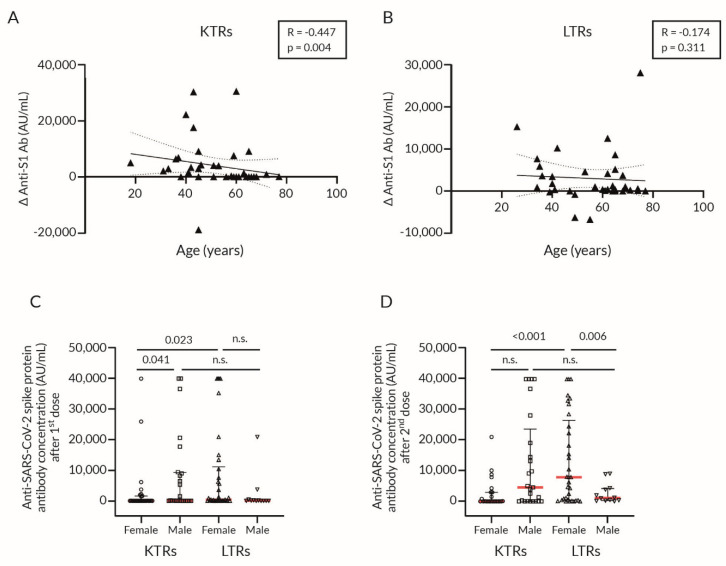
Immune response to BNT162b2 vaccination depending on the type of transplanted organ and age (**A**,**B**) or gender (**C**,**D**). Association of the Δanti-S1Ab with age in KTRs (**A**) and LTRs (**B**) was calculated with Spearman’s correlation, separate values were fitted with simple linear regression with 95% confidence interval; the scatter-dot plot (**C**,**D**) with median (middle line), lower (25%), and upper (75%) quartile (as whiskers), the *p*-value was calculated with non-parametric Mann–Whitney U Test, Δ anti-S1 Ab—difference in anti-SARS-CoV-2 spike protein antibody concentration between 1st and 2nd dose of the vaccine; 1st dose = 4–6 weeks after the first dose of vaccination; 2nd dose = 4–8 weeks after the second dose of vaccination; BNT162b2 = BioNTech/Pfizer COVID-19 mRNA vaccine; KTRs = kidney transplant recipients; LTRs = liver transplant recipients; n.s. = not significant; R—Spearman’s coefficient.

Spearman’s correlation analysis of age confirmed its significant association with the Δ anti-S1 Ab in KTRs (*p* = 0.004) (Figure 5A), but not in LTRs (*p* = 0.31) (Figure 5B). Additionally, we observed the relationship between sex and anti-S1 Ab levels. After the first dose, male KTRs produced a significantly higher amount of anti-S1 Ab than female KTRs. Furthermore, female LTRs had a better response to vaccination than female KTRs (Figure 5C,D and Table 4).

We found no correlation between number of immunosuppressive drugs taken (single-, double-, or triple-drug maintenance therapy) (Appendix A, Table A1) or blood type (Appendix A, Table A2 and Table A3) with the titer of anti-S1 Ab after the second dose of vaccine in KTRs and LTRs. In KTRs who received triple-drug maintenance IS, a significant association between gender and anti-S1 Ab titer was observed (Appendix A, Table A2). In KTRs on double-drug therapy, the time from transplantation was positively correlated with anti-S1 Ab response to vaccination (Appendix A, Table A3). In LTRs on monotherapy, the Δanti-S1 Ab was correlated with blood type (Appendix A, Table A2).

## 4. Discussion

The fundamental finding of our study was the unexpected high response rate to BNT162b2 vaccination in SOT recipients, and especially in LTRs. These results are up to date with the current worldwide discussion regarding the need for the third dose of vaccine. The response rate of an immunocompetent patient to the first dose of BNT162b2 vaccine varies between 74% and 91%, depending on the patient’s age; whereas, the second dose increases the rate of the seropositivity up to >90%, regardless of age [22]. In contrast, non-immunocompetent patients, including SOT recipients, have a much weaker humoral immune response defined by the anti-S Ab level. Of course, it depends on the type of transplanted organ and/or IS used. Studies have shown a response rate up to 37.5% of KTRs and 47.5% of LTRs following vaccination with BNT162b2 [11,23]. The success rate can be even lower, as reported by Boyarsky et al. [24], who found only 17% of patients with a positive anti-S1 Ab response after one dose of vaccine. Given such data, the average 74% positive response rate in our cumulative KTR and LTR groups was unexpected. It should be underlined that all of our patients were vaccinated at the same center, in a similar time frame, with the BNT162b2 mRNA vaccine ordered at the same time and patients were followed up homogenously by the same team of physicians. In our opinion, these factors should be considered in the discussion about the success rate of the vaccination. Both the percentage and difference between the humoral response to vaccination in our KTR (58.5%) and LTR (89.1%) groups need additional evaluation. 

It is clear that the above differences may result from the IS used and type of transplanted organ [25]. Previous studies have shown the role of the impaired immune response to any vaccination in SOT patients, most likely because of the IS therapy [9]. The suppression of humoral immunity (e.g., by rituximab or methotrexate) in non-SOT patients can reduce the production of neutralizing Ab in response to other vaccines [26]. It is worth noting that the liver is an immunologically privileged organ, and a better response to vaccination by LTRs may be a result of this physiologic phenomenon. LTRs usually require less IS than KTRs and are more likely to be on a one- or two-drug maintenance therapy. Interestingly, our results did not reveal significant differences between the effect of two- or three-drug IS on the response to vaccination. The time from transplantation to vaccination also did not influence the response; however, TAC dose at vaccination correlated with anti-S1 Ab serum concentration after both doses. These observations suggest that IS intensity at the moment of vaccination (related to the type of transplanted organ), rather than its serum concentration may impact the response to vaccination. 

The future knowledge about the impact of genetic background on humoral responses will help understanding interpersonal differences and could enable personalized approach to vaccinations. As LTRs usually require less IS than KTRs and repeatedly show superior response to vaccination compared to other SOT recipients, including KTRs [12], the intensity of IS may be a contributing factor. Furthermore, the IS protocol for LTRs used in our center allows its minimization in individuals with no autoimmune primary liver disease. This could influence the response to the vaccination; however, the number of patients in our study was insufficient to prove such influence. Therefore, larger population studies are necessary to answer this question. We found that anti-S1 Ab titers were associated with patients’ age, which is in line with the results of a recent study performed in the healthy population. Authors shown that BNT162b2 vaccine was effective in producing anti-SARS-CoV-2 IgG levels, and that humoral response was age- and gender-dependent [27]. However, in our case, only female LTRs responded better comparing to KTRs. We do not fully understand this phenomenon that definitely needs to be explained in a separate study.

The unexpected finding was the average anti-S1 Ab titer >40.000 AU/mL after the second dose in the LTR group with autoimmune primary liver diseases (primary sclerosing cholangitis or autoimmune hepatitis), which confirmed previous observations that autoimmunity itself does not hamper vaccination responses [28]. It is unclear whether the Abs are the crucial and only indicator of immunity or whether their absence leads to a complete lack of response to vaccination. Furthermore, people who have received a full course of vaccination due to cross-neutralization of at least some of the circulating SARS-CoV-2 variants have a positive response to the new SARS-CoV-2 variants [29]. The question is raised of whether recipients who have received vaccination, despite the lack of Ab production, may also have such a response to the new SARS- CoV-2 variants.

A very important issue emerged from the publication of the Krause et al. [30], who discussed the need of the third dose by the general population. Their viewpoint is that two doses seem to be sufficient and there are known risks of side effects. Therefore, boosting should be considered only if there is a clear evidence that it is appropriate. Previous studies have indicated that patients vaccinated with a third dose of SARS-CoV-2 vaccine presented with stronger immunity, were less likely to get infected, and had milder symptoms of SARS-CoV-2 infection [31,32]. The viewpoint of Krause and colleagues [30] are particularly important for non-immunocompetent patients. Among them, SOT patients experience many adverse effects due to the polytherapy, and their response to the vaccination is less predictable. Therefore, it seems all the more reasonable to refrain from administering a third dose of the vaccine to this patient population, particularly in the context of a possible high response rate to the first and second doses, as presented in our study.

It is possible that SOT patients need an individualized vaccination algorithm. This may include more than two doses in selected patients with a lower response to the first and second doses [10], or even a combined scheme with mRNA vaccines, protein/subunit vaccines, and vector-based vaccines [33,34]. We believe that patients who have produced no anti-S1 Ab after the second dose of vaccine should be candidates for additional immune testing in order to choose an optimal vaccination scheme. 

Lastly, the socioeconomic status and geographic factors differentiating Poland from other European countries were recently discussed in relation to nearly ten times lower SARS-CoV-2 seroprevalence after the first wave of COVID-19 pandemic [35]. We think that genetic predispositions together with IS intensity and geographic factors could partially explain different from previously reported response to vaccination in our population.

SARS-CoV-2 vaccinations commenced in January 2021; therefore, we currently have a maximum of 6-month vaccine efficacy data available [36]. The BNT162b2 vaccine has a favorable safety profile and is highly efficacious in preventing COVID-19 in the short term. However, we do not know the long-term effects of vaccination, especially in non-immunocompetent patients including SOT recipients. Thus, follow-up of patients is needed for the next several years.

The current studies on post-vaccination response should provide a background to understand the immunology and biology of the COVID-19. Such results already have an impact on both, prevention and treatment strategies. The investigations to determine and verify the specific subtype of integrins to interact with the S SARS-CoV-2 protein are an example. The RGD-related peptides are currently being used in clinical trials, since blocking these interactions of the RGD motif may serve as a potential drug candidate for COVID-19 [37,38].

## 5. Conclusions

In conclusion, we showed a higher than previously reported humoral response to two vaccine doses, especially in LTRs, confirming that the majority of SOT recipients can achieve a sufficient level of serum anti-S1 Ab. The response to vaccination depends on many factors of which the patient’s age, type of transplanted organ, and IS used seem to be most important. We also confirmed the safety of BNT162b2 vaccination in KTRs and LTRs. Additional clinical observations and basic research are needed to define the background of the variable vaccine response in immunocompromised patients, including transplant recipients.

## Figures and Tables

**Table 1:** Demographic and clinical characteristics of study participants *.

Characteristics	KTRs (*n* = 61)	LTRs (*n* = 55)
Mean age (SD), years	54.4 (12.9)	58.4 (13.3)
Median age (Range), years		
Female	61 (31–77)	60 (26–82)
Male	53.5 (18–71)	65 (50–71)
Gender, *n* (%)		
Female	33 (54.1)	21 (43.6)
Male	28 (45.9)	44 (80)
Mean BMI (SD), kg/m^2^	25.1 (3.9)	25.7 (4.0)
Mean time since transplantation (SD), years	13 (7.1)	14.8 (3.8)
History of COVID-19 infection, *n* (%)		
Infection confirmed by PCR	5 (8.2)	5 (9.1)
Hospitalization due to COVID-19	3 (60) ^†^	1 (20) ^†^
Symptoms:		
Fever >38 °C	3 (60)	4 (80)
Loss of smell and/or taste	0 (0)	3 (60)
Dyspnea	2 (40)	2 (40)
Sore throat	1 (20)	1 (20)
Myalgia	2 (40)	3 (60)
Cough	2 (40)	2 (40)
Pneumoniae	2 (40)	2 (40)
Tachycardia/arrythmia	0	3 (40)
Diarrhea	1 (20)	3 (40)
Other	1 (20)	2 (40)
Induction therapy, *n* (%)		
Anti-thymocyte globulin	0	0
Anti-interleukin-2 receptor	0	30 (54.5)
Immunosuppression, *n* (%)		
Steroids	52 (85.2)	20 (36.4)
Mycophenolate mofetil	47 (77.1)	16 (29.1)
Azathioprine	7 (11.5)	5 (9.1)
Cyclosporine	25 (41)	11 (20)
Tacrolimus	33 (54.1)	43 (78.2)
Sirolimus	2 (3.3)	2 (3.6)
Everolimus	1 (1.6)	2 (3.6)
Immunosuppression, *n* (%)		
Mono-therapy (CNI/MMF)	0	24 (43.6)
Dual-therapy (CNI + GKS/MMF/AZA/mTORI)	16 (26.3)	18 (32.7)
Triple-therapy (CNI/mTORi +GKS + MMF/AZA)	45 (73.8)	13 (23.6)
Mean laboratory data (SD)		
Serum creatinine, mg/dL	1.4 (0.5)	1 (0.3)
eGFR, mL/min * 1.73 m^2^	51.3 (16.8)	58.4 (19.1)
ALT, IU/L	18.6 (10.5)	25.1 (17.7)
AST, IU/L	n.a.	26.4 (15.4)
GGTP, IU/L	n.a.	70 (78.3)
ALP, IU/L	n.a.	109.4 (65.4)
Bil, mg/dL	n.a.	1.3 (2.4)
Complement component		
C3 G/L	1.2 (0.3)	1.3 (0.3)
C4 G/L	0.2 (0.1)	0.2 (0.1)

* Parameters determined after 4–8 weeks after the second dose of BNT162b2 vaccination. ^†^ Percent of infection confirmed by PCR. ALP = alkaline phosphatase; ALT = alanine aminotransferase; AST = aspartate aminotransferase; AZA = azathioprine; Bil = bilirubin; BMI = body mass index; BNT162b2 = BioNTech/Pfizer COVID-19 mRNA vaccine; CNI = calcineurin inhibitor including cyclosporine and tacrolimus; Cr = creatinine; GFR = glomerular filtration rate (estimated with CKD-EPI formula); GGTP = gamma-glutamyltranspeptidase; GKS = corticosteroids; KTRs = kidney transplant recipients; LTRs = liver transplant recipients; MMF = mycophenolate mofetil; mTORi = mTOR kinase inhibitors including everolimus and sirolimus; n.a. = not available.

**Table 2:** Comparison of SARS-CoV-2 spike protein Ab concentration (AU/mL) after BNT162b2 vaccination in KTRs and LTRs.

Parameter	KTRs + LTRs (*n*)	MD (IQR)	KTRs (*n*)	MD (IQR)	LTRs (*n*)	MD (IQR)	*p*-Value *
All patients: positive history of COVID-19 disease (confirmed by PCR test) included							
Δ anti-S1 Ab ^†^	76	725.4 (4434.4)	40	604 (4635.6)	36	730.1 (4384)	0.84
Ab after the 2nd dose	94	2238.2 (10,475.2)	49	860 (9198.9)	45	4351 (15,024.7)	0.032
Ab after the 1st dose	98	73.2 (5639.9)	52	39.1 (4549)	46	231.7 (6192)	0.197
Patients without a positive history of COVID-19 disease							
Δ anti-S1 Ab ^†^	69	402.1 (4178)	36	55.4 (4512.1)	33	869.4 (4037)	0.477
Ab after the 2nd dose	87	1210.2 (8910.5)	45	99.5 (5077.6)	42	3193.55 (10,112)	0.017
Ab after the 1st dose	88	48.8 (1788.8)	47	23.8 (1945.3)	41	148 (1263.3)	0.266

*n*—number of observations; values are set as median (MD) and interquartile range (IQR); * Mann–Whitney test; *p* < 0.05 was considered statistically significant (comparison of variables in a group of KTRs and LTRs); ^†^ difference between anti-SARS-CoV-2 spike protein antibody titer after the first and second doses of BNT162b2 vaccine; 1st dose = 4–6 weeks after the first dose of vaccination; 2nd dose = 4–8 weeks after the second dose of vaccination; Ab = SARS-CoV-2 spike protein antibody; BNT162b2 = BioNTech/Pfizer COVID-19 mRNA vaccine; KTRs = kidney transplant recipients; LTRs = liver transplant recipients.

**Table 3:** Correlations of SARS-CoV-2 spike protein Ab concentration (AU/mL) after BNT162b2 vaccination with clinical variables.

Parameter	All Patients			KTRs			LTRs		
	*n*	*p*-Value *	R	*n*	*p*-Value *	R	*n*	*p*-Value *	R
Correlations with Δ anti-S1 Ab ^†^									
Anti-N Ab ^‡^, S/CO	75	0.103	0.190	40	0.43	0.006	35	0.64	−0.082
Age, years	76	0.005	−0.317	40	0.004	−0.447	36	0.31	−0.174
BMI, kg/m^2^	76	0.071	−0.208	40	0.29	−0.170	36	0.148	−0.246
Time from transplantation, years	76	0.87	0.020	40	0.99	0.002	36	0.80	−0.043
CSA									
daily dose, mg	21	0.70	0.089	13	0.74	0.100	8	0.47	0.299
Concentration ^‡^, ng/mL	21	0.48	−0.163	13	0.61	−0.156	8	0.74	−0.143
TAC									
daily dose, mg	53	0.22	0.171	25	0.50	0.142	28	0.37	0.200
Concentration ^‡^, ng/mL	52	0.30	−0.148	24	0.46	−0.158	28	0.23	−0.233
Graft function									
Cr, mg/dL	65	0.26	−0.141	37	0.59	−0.090	28	0.145	−0.282
GFR, mL/min × 1.73 m^2^	66	0.036	0.259	37	0.102	0.273	29	0.35	0.179
AST, IU/L	67	0.41	0.102	34	0.82	0.039	33	0.124	0.273
ALT, IU/L	37	0.56	0.100	5	0.39	−0.500	32	0.32	0.183
ALP, IU/L	35	0.41	0.144	4	0.37	−0.632	31	0.31	0.189
Bil, mg/dL	35	0.51	−0.115	3	n.a.	n.a.	32	0.79	−0.050
GGTP, IU/L	30	0.115	−0.294	1	n.a.	n.a.	29	0.188	−0.252
Complement component, G/L									
C3	56	0.54	−0.084	30	0.96	0.010	26	0.25	−0.235
C4	55	0.26	−0.155	30	0.52	−0.123	25	0.43	−0.166
Correlations with anti-S1 Ab after the 2nd dose									
Anti-N Ab ^§^, S/CO	35	0.040	0.409	25	0.106	0.331	20	0.41	0.194
Age, years	94	0.014	−0.253	49	<0.001	−0.467	45	0.62	−0.076
BMI, kg/m^2^	94	0.74	0.034	49	0.77	0.042	45	0.96	0.008
Time from transplantation, years	94	0.32	0.104	49	0.25	0.169	45	0.25	−0.174
CSA									
daily dose, mg	30	0.27	0.209	20	0.062	0.425	10	0.45	0.271
Concentration ^§^, ng/mL	28	0.81	0.049	20	0.63	0.114	8	0.46	0.310
TAC									
daily dose, mg	62	0.119	0.200	27	0.69	0.080	35	0.040	0.349
Concentration ^§^, ng/mL	57	0.180	−0.180	26	0.87	0.033	31	0.162	−0.257
Correlations with anti-S1 Ab after the 1st dose									
Anti-N Ab ^‡^, S/CO	97	<0.001	0.671	52	<0.001	0.632	45	<0.001	0.711
Age, years	97	<0.001	−0.346	52	<0.001	−0.462	45	0.139	−0.224
BMI, kg/m^2^	94	0.86	0.018	50	0.51	0.096	44	0.86	−0.027
Time from transplantation, years	94	0.181	0.139	49	0.23	0.173	45	0.96	−0.007
CSA									
daily dose, mg	27	0.188	0.261	18	0.33	0.245	9	0.24	0.434
Concentration ^‡^, ng/mL	27	0.088	0.335	18	0.28	0.270	9	0.081	0.610
TAC									
daily dose, mg	67	0.057	0.234	31	0.163	0.257	36	0.26	0.194
Concentration ^‡^, ng/mL	65	0.56	−0.074	29	0.68	−0.080	36	0.38	−0.152

*n*—number of observations for selected pairs of parameters. * Spearman’s test; *p* < 0.05 was considered statistically significant (comparison of variables in a group all participants, KTRs and LTRs). ^†^ Difference between anti-SARS-CoV-2 spike protein antibody titer after the first and second doses of BNT162b2 vaccination; ^‡^ concentration 4–6 weeks after the first dose of vaccination; ^§^ concentration 4–8 weeks after the second dose of vaccination; 1st dose = 4–6 weeks after the first dose of vaccination; 2nd dose = 4–8 weeks after the second dose of vaccination; ALP = alkaline phosphatase; ALT = alanine aminotransferase; anti-N Ab = anti-SARS-CoV-2 nucleocapsid protein antibody associated with a reaction to previous contact with the SARS Cov-2 virus; anti-S1 Ab = SARS-Cov-2 spike protein antibody; AST = aspartate aminotransferase; BMI = body mass index; BNT162b2 = BioNTech/Pfizer COVID-19 mRNA vaccine; Cr = creatinine; CSA = Cyclosporine; GFR = glomerular filtration rate (estimated with CKD-EPI formula); GGTP = gamma-glutamyltranspeptidase; KTRs = kidney transplant recipients; LTRs = liver transplant recipients; n.a. = not available; R–spearman’s coefficient; TAC = tacrolimus.

**Table 4:** Comparison of SARS-CoV-2 spike protein Ab concentration (AU/mL) after BNT162b2 vaccination with clinical variables.

Parameter	All Patients		KTRs		LTRs		
	*n*	MD (IQR)	*n*	MD (IQR)	*n*	MD (IQR)	*p*-Value *
Comparison to Δ anti-S1 Ab ^†^							
Gender							
M	30	2509 (7408.8)	21	3398.7 (9104.1)	9	1006.7 (3826.6)	0.86
F	46	251.5 (3476.8)	19	10.4 (2931.4)	27	590.7 (4612.2)	0.35
Blood type							
A	29	402.1 (4177.5)	16	55 (3164.8)	13	869.4 (7567.6)	0.32
B	13	351.4 (2974.3)	5	1333.3 (2974.3)	8	291.7 (2769.9)	0.88
AB	8	1485.7 (6572.6)	6	3060.5 (8275.5)	2	−389.6 (779.1)	0.094
O	24	1147.2 (4813.4)	11	0 (6878.2)	13	1236.7 (3348.5)	0.64
Comparison to Ab after the 2nd dose							
Gender							
M	37	2583.7 (9723.5)	25	4587.3 (19,037.8)	12	1128.5 (3824)	0.26
F	57	1511.5 (10,487.1)	24	12.1 (2996.3)	33	7928 (24,050.8)	<0.001
Comparison to Ab after the 1st dose							
Gender							
M	36	107.9 (7375.3)	24	119.7 (9196.1)	12	81.2 (304.5)	0.45
F	62	68.3 (3753.2)	28	2.7 (1560.5)	34	390.7 (10,404.8)	0.023

*n* = number of observations; values are set as median (MD) and interquartile range (IQR); * Mann–Whitney test; *p* < 0.05 was considered statistically significant (comparison of KTRs and LTRs); ^†^ difference between anti-SARS-CoV-2 spike protein antibody titer after the first and second doses of BNT162b2 vaccine; 1st dose = 4–6 weeks after the first dose of vaccination; 2nd dose = 4–8 weeks after the second dose of vaccination; Ab = SARS-CoV-2 spike protein antibody; BNT162b2 = BioNTech/Pfizer COVID-19 mRNA vaccine; KTRs = kidney transplant recipients; LTRs = liver transplant recipients; F = female, M = male.

## Data Availability

Data available on request due to privacy restrictions, the data presented in this study are available on request from the corresponding author.

## Appendix A

**Table A1:** Correlation of SARS-CoV-2 spike protein Ab concentration (AU/mL) after BNT162b2 vaccination to maintenance immunosuppression in KTRs and LTRs.

	Mono-Therapy		Double-Therapy		Triple-Therapy		*p*-Value
	*n*	MD (IQR)	*n*	MD (IQR)	*n*	MD (IQR)	
KTRs							
Δ anti-S1 Ab ^‡^	n.a.	n.a.	10	55.4 (1619.3)	30	1722.3 (5014.6)	0.54 *
Ab after 2nd dose	n.a.	n.a.	11	99.5 (19,056.5)	38	1434.3 (8823.5)	0.52 *
Ab after 1st dose	n.a.	n.a.	15	49.6 (3753.2)	37	36.0 (5344.7)	0.79 *
LTRs							
Δ anti-S1 Ab ^‡^	17	1006.7 (4380.2)	10	274.7 (3611.4)	10	1412.5 (7708.6)	0.76 ^†^
Ab after 2nd dose	20	4132.8 (12,768.8)	14	4967.0 (21,904.5)	12	6155.2 (21,904.5)	0.98 ^†^
Ab after 1st dose	21	314.7 (3538.2)	14	15.7 (10,404.8)	11	263.0 (6192.0)	0.68 ^†^

*n*—number of observations; values are set as median (MD) and interquartile range (IQR); * Mann–Whitney test; *p*-value < 0.05 was considered significant (comparison of variables in a group double-therapy and triple-therapy); ^†^ Kruskal–Wallis test; (*p*-value < 0.05 was considered significant (comparison of variables in a group mono-therapy, double-therapy and triple-therapy); ^‡^ difference between anti-SARS-CoV-2 spike protein antibody titer after the first and the second dose of BNT162b2 vaccine; 1st dose = 4–6 weeks after the first dose of vaccination; 2nd dose = 4–8 weeks after the second dose of vaccination; Ab = SARS-CoV-2 spike protein antibody; BNT162b2 = BioNTech/Pfizer COVID-19 mRNA vaccine; KTRs = kidney transplant recipients; LTRs = liver transplant recipients; n.a. = not available.

**Table A2:** Associations of SARS-CoV-2 spike 1 protein Ab concentration (AU/mL) after BNT162b2 vaccination with demographic and clinical variables in KTRs and LTRs depending on the maintenance immunosuppression.

Parameter	Sex						Blood Type										Autoimmune vs. Non-Autoimmune Cause of Liver Transplantation		
Group	KTRs			LTRs			KTRs					LTRs					LTRs		
Mono-therapy																			
Variable	M (*n* = 0)	F (*n* = 0)	*p*-value *	M (*n* = 10)	F (*n* = 14)	*p*-value *	A (*n* = 0)	B (*n* = 0)	AB (*n* = 0)	O (*n* = 0)	*p*-value ^†^	A (*n* = 4)	B (*n* = 8)	AB (*n* = 0)	O (*n* = 11)	*p*-value ^†^	Yes (*n* = 3)	No (*n* = 21)	*p*-value *
Δ anti-S1 Ab ^‡^	n.a.	n.a.	n.a.	2370.4 (3826.6)	590.7 (4684.3)	0.65	n.a.	n.a.	n.a.	n.a.	n.a.	2384.4 (9504.6)	232 (351.4)	n.a.	4173.1 (4180.3)	0.031	1236.7 (27,619.1)	684.35 (4612.2)	0.34
Ab after 2nd dose	n.a.	n.a.	n.a.	1593.3 (3372.5)	7116.2 (28,621.3)	0.132	n.a.	n.a.	n.a.	n.a.	n.a.	6726.9 (21,715.9)	1210.2 (32,756.8)	n.a.	3887.1 (4328.1)	0.53	2500 (31,223.5)	4378.4 (8028.6)	1
Ab after 1st dose	n.a.	n.a.	n.a.	137.8 (275.8)	1034.9 (7418)	0.180	n.a.	n.a.	n.a.	n.a.	n.a.	307.9 (17,748)	94.3 (7566)	n.a.	349.8 (3456.4)	0.59	1263 (3604.4)	257.55 (7499.8)	1
Double-therapy																			
Variable	M (*n* = 6)	F (*n* = 10)	*p*-value *	M (*n* = 4)	F (*n* = 14)	*p*-value *	A (*n* = 4)	B (*n* = 4)	AB (*n* = 1)	O (*n* = 6)	*p*-value ^†^	A (*n* = 7)	B (*n* = 3)	AB (*n* = 2)	O (*n* = 5)	*p*-value ^†^	Yes (*n* = 8)	No (*n* = 9)	*p*-value *
Δ anti-S1 Ab ^‡^	1619.3 (17,595.2)	11.3 (99.5)	0.30	2662.5 (5030.4)	271.6 (2240.4)	0.51	52.2 (856.9)	8791.5 (17,617.4)	n.a.	11.3 (49,526.5)	0.96	869.4 (3231.6)	n.a.	n.a.	84.8 (125)	0.088	144.15 (193.1)	2251.6 (5177.7)	0.46
Ab after 2nd dose	4969.1 (19,037.8)	99.5 (846.3)	0.65	1013 (8766.7)	14160.4 (24,085.9)	0.53	52.3 (4986.1)	9551.3 (19,037.8)	n.a.	10560.6 (30,493.3)	0.61	9555.3 (21,904.5)	8920.9 (33,722.2)	n.a.	154.2 (414.9)	0.118	402.1 (22,165.6)	14,160.4 (23,475)	0.096
Ab after 1st dose	1470 (8208.8)	2.7 (1632.3)	0.24	6.9 (3743.2)	24.5 (14,939.5)	0.69	0.015 (4129.2)	62.8 (748.2)	n.a.	2692.8 (9409.9)	0.21	2832.2 (10,404.8)	n.a.	n.a.	0 (3.5)	0.088	0 (6.9)	4691.6 (17,884.7)	0.153
Triple-therapy																			
Variable	M (*n* = 22)	F (*n* = 23)	*p*-value *	M (*n* = 1)	F (*n* = 12)	*p*-value *	A (*n* = 15)	B (*n* = 5)	AB (*n* = 8)	O (*n* = 16)	*p*-value ^†^	A (*n* = 5)	B (*n* = 3)	AB (*n* = 0)	O (*n* = 5)	*p*-value ^†^	Yes (*n* = 10)	No (*n* = 3)	*p*-value *
Δ anti-S1 Ab ^‡^	3704.2 (8330.1)	5.5 (2974.3)	0.094	n.a.	1767.3 (7708.6)	n.a.	347.9 (4256)	1333.3 (2974.3)	4009.6 (7024.2)	0 (5014.6)	0.64	3854.3 (12,117.7)	n.a.	n.a.	1412.5 (1900.5)	0.91	1767.3 (7708.6)	385.5 (10,234.5)	0.91
Ab after 2nd dose	4587.3 (28,130.5)	3.7 (3016.5)	0.012	n.a.	7959.3 (15130)	n.a.	347.9 (13,235.4)	1.1 (1357.1)	4454.2 (10,470.6)	779.9 (8823.5)	0.20	10497.5 (25,602.7)	296.8 (15,426.8)	n.a.	2728.3 (5409.6)	0.29	7959.3 (14,321.2)	385.5 (10,497.5)	0.36
Ab after 1st dose	93.4 (17,725.7)	1.8 (1488.6)	0.098	n.a.	329.8 (6144)	n.a.	4 (8979.4)	23.8 (42.2)	2209.8 (17,725.7)	33.3 (1045.7)	0.77	20198.3 (39,670.3)	n.a.	n.a.	48 (874.2)	0.139	635.4 (23,036.8)	0 (263)	0.122

*n*—number of patients in selected subgroups; values are set as the median (MD) and interquartile range (IQR); * Mann–Whitney test. *p* < 0.05 was considered statistically significant; ^†^ Kruskal–Wallis test; *p* < 0.05 was considered statistically significant; ^‡^ difference between anti-SARS-CoV-2 spike protein antibody titer after the first and second doses of BNT162b2 vaccine; 1st dose = 4–6 weeks after the first dose of vaccination; 2nd dose = 4–8 weeks after the second dose of vaccination; Ab = Sars-anti-SARS-CoV-2 spike protein antibody; BNT162b2 = BioNTech/Pfizer COVID-19 mRNA vaccine; F = female; KTRs = kidney transplant recipients; LTRs = liver transplant recipients; M = male; n.a. = not available.

**Table A3:** Associations of SARS-CoV-2 spike protein Ab concentration (AU/mL) after BNT162b2 vaccination with BMI and time from transplantation in KTRs and LTRs depending on the number of immunosuppressive drugs.

Parameter	BMI						Time from Transplantation					
Group	KTRs			LTRs			KTRs			LTRs		
Variable	*n*	*p*-Value *	R	*n*	*p*-Value *	R	*n*	*p*-Value *	R	*n*	*p*-Value *	R
Mono-therapy												
Δ anti-S1 Ab ^†^	n.a.	n.a.	n.a.	17	0.48	−0.184	n.a.	n.a.	n.a.	17	0.40	0.217
Ab after 2nd dose	n.a.	n.a.	n.a.	20	0.64	−0.111	n.a.	n.a.	n.a.	20	0.37	0.213
Ab after 1st dose	n.a.	n.a.	n.a.	20	0.46	−0.176	n.a.	n.a.	n.a.	21	0.50	0.154
Double-therapy												
Δ anti-S1 Ab ^†^	10	0.73	−0.127	10	0.83	−0.079	10	0.37	0.321	10	0.75	−0.117
Ab after 2nd dose	11	0.50	−0.227	14	0.081	0.481	11	0.049	0.603	14	0.163	−0.394
Ab after 1st dose	14	0.60	−0.153	13	0.62	0.153	13	0.019	0.639	13	0.54	−0.188
Triple-therapy												
Δ anti-S1 Ab ^†^	30	0.48	−0.134	10	0.062	−0.608	30	0.84	−0.038	10	0.51	−0.238
Ab after 2nd dose	38	0.70	0.064	12	0.059	−0.559	38	0.77	0.049	12	0.173	−0.421
Ab after 1st dose	36	0.46	0.126	11	0.43	−0.267	36	0.66	0.076	11	0.84	0.067

*n*—number of observations for selected pairs of parameters; * Spearman’s test *p* < 0.05 was considered statistically significant (comparison of variables in a group all participants, KTRs and LTRs); ^†^ difference between anti-SARS-CoV-2 spike protein antibody titer after the first and second doses of BNT162b2 vaccine; 1st dose = 4–6 weeks after the first dose of vaccination; 2nd dose = 4–8 weeks after the second dose of vaccination; Ab = SARS-CoV 2 spike 1 protein antibody; BMI = body mass index; BNT162b2 = BioNTech/Pfizer COVID-19 mRNA vaccine; KTRs = kidney transplant recipients; LTRs = liver transplant recipients; n.a. = not available.

**Table A4:** The results of anti-SARS-CoV-2 nucleocapsid protein Ab assays in all patients regardless of con-firmed COVID-19.

	KTRs		LTRs		*p*-Value *
Anti-N Ab S/CO	*n*	MD (IQR)	*n*	MD (IQR)	
After 1st dose	53	0.06 (0.26)	45	0.05 (0.77)	0.946
After 2nd dose	25	0.0 (0.0)	21	0.0 (0.31)	0.067

*n*—number of observations; values are set as the median (MD) and interquartile range (IQR); * Mann–Whitney U Test, *p* < 0.05 was considered statistically significant; 1st dose = 4–6 weeks after the first dose; 2nd dose = 4–8 weeks after the second dose; Anti-N Ab = anti-SARS-CoV-2 nucleocapsid protein antibody; BNT162b2 = BioNTech/Pfizer COVID-19 mRNA vaccine; KTRs = kidney transplant recipients; LTRs = liver transplant recipients; S/CO—signal/cut off.
